# Landscape- and Local-Scale Actions Are Essential to Conserve Regional Macrophyte Biodiversity

**DOI:** 10.3389/fpls.2018.00599

**Published:** 2018-05-17

**Authors:** Munemitsu Akasaka, Shinsuke Higuchi, Noriko Takamura

**Affiliations:** ^1^Institute of Agriculture, Tokyo University of Agriculture and Technology, Fuchu, Japan; ^2^Department of Biology, Graduate School of Science, Kobe University, Kobe, Japan; ^3^National Institute for Environmental Studies, Tsukuba, Japan

**Keywords:** aquatic plants, beta diversity, community assemblage, irrigation pond, regional conservation planning, topographic wetness index

## Abstract

Regional-scale pond diversity is supported by high variation in community composition. To effectively and efficiently conserve pond regional diversity, it is essential to recognize the community types in a focal region and the scales of the factors influencing the occurrence of respective community types. Based on a flora survey and GIS analysis of 367 ponds in western Japan, we developed a multinomial regression model that describes the relationship between aquatic macrophyte community type (based on cluster analysis) and five environmental factors that differ in the spatial scale at which they operate (i.e., landscape or local scale) and origin (i.e., natural or anthropogenic). A change in topographic configuration resulted in a transition of the community types with high species richness. Increasing urban and agricultural area around ponds resulted in a decrease in species-rich community occurrence; an increase in urban area increased the probability of a pond having no macrophytes, whereas that of paddy field increased the probability of a pond having only a few macrophytes. Pond surface area and proportion of artificial embankment significantly defined the pond community: greater embankment proportions increased the probability of ponds having few or no macrophytes. Our results suggest that conserving regional pond biodiversity will require actions not only at a local scale but also at a sufficiently large spatial scale to cover the full gradient of topographic configurations that influence the macrophyte species composition in ponds.

*We dedicate this paper to the memory of our co-author SH, who has sadly passed away*.

## Introduction

Both natural and man-made ponds are important habitats in the context of regional freshwater biodiversity conservation, because they vary greatly in species composition and contain unique species (Kadono, 1998; Williams et al., 2004; Davies et al., 2008). As in other freshwater habitats, however, pond biodiversity has been rapidly declining because of anthropogenic pressures, including urbanization of the surrounding land, habitat loss, and eutrophication (Bronmark and Hansson, 2002; Ishii and Kadono, 2003; Akasaka et al., 2010; Kadoya et al., 2011; Stendera et al., 2012; Takamura, 2012; Toyama and Akasaka, 2017). To address this decline, there is a growing body of research focusing on pond biodiversity conservation, although the focus is more on conservation of α diversity (e.g., Akasaka et al., 2010; Akasaka and Takamura, 2011) than on conservation of regional pond diversity (Oertli et al., 2002; Akasaka and Takamura, 2012; Lukács et al., 2013).

Regional biodiversity (i.e., γ diversity) is composed of species richness within a pond (α diversity) and diversity among ponds (β diversity), and β diversity is expressed as the variation in species composition among ponds (Clarke et al., 2010; Akasaka and Takamura, 2012). Although the relative contributions of α and β diversity to γ diversity differ depending on focal ecosystems and taxa (Cottenie, 2005), high variation in species composition supports γ diversity of pond-dwelling species, such as submerged and floating-leaved macrophytes, aquatic macroinvertebrates, and fishes (Williams et al., 2004; Hassall et al., 2011; Mitsuo et al., 2011; Akasaka and Takamura, 2012; Lukács et al., 2013); in other words, ensuring variation in species composition is key to conserving regional pond biodiversity. Although practical approaches for ensuring variation in species composition have rarely been addressed, conserving distinct community types within a region has been shown to be effective (Trakhtenbrot and Kadmon, 2005; Castillo-Campos et al., 2008). With this approach in mind, one practicable step for conserving a wide variation in species composition would be to identify community types and the environmental conditions that support the respective community types, although this has not yet been addressed.

The species composition in a pond is influenced by natural environmental factors at a landscape scale, such as elevation and topographic configuration, and by those at a local scale, such as pond surface area, which serves as a proxy for habitat extent and water retention capacity (e.g., Larson et al., 2009). In addition, species composition is also affected by anthropogenic pressures (Cheruvelil and Soranno, 2008; Toyama and Akasaka, 2017) corresponding to the landscape scale, such as the degree of urbanization of the surrounding area, and/or the local scale, such as the proportion of the pond’s perimeter protected by concrete embankments. For pond biodiversity conservation, therefore, it seems clear that both natural and anthropogenic factors need to be considered at both the landscape and local scales (Toivonen and Huttunen, 1995; Declerck et al., 2006; Rolon et al., 2012). However, the relative importance of natural and anthropogenic factors on pond community composition at these scales has rarely been evaluated explicitly, although there have been many such studies on lakes (Capers et al., 2010; Alahuhta et al., 2013), in which ecological processes differ from those in ponds (Céréghino et al., 2008 and references therein).

Gaining such an understanding could provide important information to support biodiversity conservation as well as insights into the forces responsible for the community structure (Cottenie, 2005). For example, when determining which ponds to conserve, ponds should be selected from across the landscape if landscape-scale factors are highly important in determining the variation of species composition, whereas they can be selected from a particular location if these factors are less important. Likewise, elucidating the influential anthropogenic pressures and their spatial scales provides important information for determining the responsible conservation agencies. National or regional governments would have greater responsibility when anthropogenic pressure at a landscape scale greatly influences the species composition, whereas the role of a pond manager or a local conservation group might increase when the pressure at a local scale is more important.

In this study, we focused on aquatic macrophytes because they provide suitable habitats to various animals (Scheffer, 2004; Bilton et al., 2006; Santi et al., 2010; Iwai et al., 2017) and because of their conservation status (Williams et al., 2004). Indeed, about 40% (269 species) of the aquatic macrophytes listed by Kadono (2014), the most comprehensive guide to Japanese aquatic macrophytes, are now registered as endangered species on the national Red List. We determined the relative importance of three landscape-scale factors (topographic configuration, and the proportions of urban area and paddy field surrounding the pond) and two local factors (pond surface area and proportion of natural shoreline covered by artificial embankment) that could influence community composition in ponds. We also assessed the effect of the five factors on the occurrence of ponds without macrophytes, because distinguishing ponds with or without macrophytes will help to narrow the conservation targets. Our aim was to evaluate the relative importance of these five factors in determining the macrophyte community in ponds. Note that our findings will help to guide regional strategies for conserving macrophyte diversity in ponds, but not to predict the macrophyte communities in ponds.

## Materials and Methods

### Study Area

**FIGURE 1:**
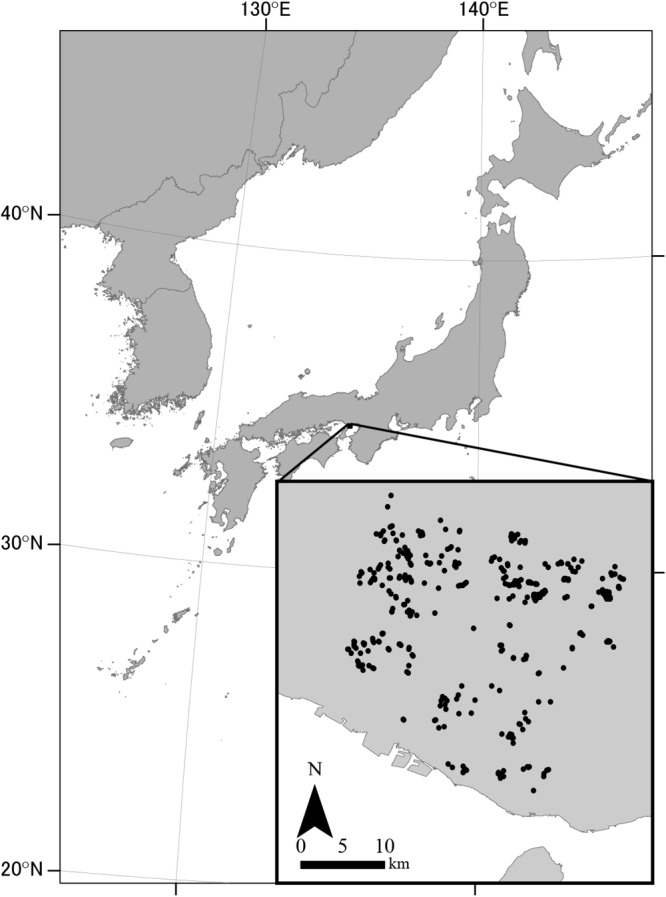
Study area in Hyogo Prefecture, Japan. Dots represent survey ponds.

We conducted our study in southwestern Hyogo Prefecture, Japan (**Figure 1**). We selected this area because of its abundant ponds (>5000 ponds within ∼780 km^2^) and the increasing interest in conserving regional freshwater biodiversity (Akasaka et al., 2010). The climate is warm and temperate, with a mean annual temperature of 14.4°C (mean minimum, 3.5°C in January; mean maximum, 26.4°C in August) and a mean annual precipitation of 1183 mm based on data at the Miki Climatological Observatory, which is located within the study area at 145 m a.s.l. The elevation range of the study area is <160 m and it generally decreases from the north to the south.

Irrigation ponds, which account for virtually all the existing ponds in this region, were created extensively before the 1910s to provide water for rice cultivation (Uchida, 2003), and more than 8% of the ponds have been lost in the last half century because of urbanization, residential development, or abandonment of agriculture (Uchida, 2003). The macrophyte biodiversity of the remaining ponds has decreased drastically, especially after the 1980s (Ishii and Kadono, 2003). In our previous studies of ponds in this region, surface area ranged from 107 to 29,204 m^2^ and potential maximum depth from 1.1 to 8.1 m (Akasaka et al., 2010; Akasaka and Takamura, 2011, 2012), although the majority of the ponds in those studies do not overlap with the ponds surveyed here.

### Macrophyte and Water Quality Survey

We haphazardly chose 369 irrigation ponds for our study (**Figure 1**). The selection of ponds was not strictly randomized; we selected ponds based on ease of access and with of a variety of sizes and characteristics. We surveyed each pond for macrophyte occurrence using an inflatable boat and a rake at the approximate time of peak macrophyte biomass (late August to September) in either 2006 or 2008. We recorded all species of floating-leaved, submerged, and free-floating submerged macrophytes using the nomenclature of Kadono (2014).

To describe the water quality for each macrophyte community type (for details, see section “Classification of the Macrophyte Communities in Ponds”), we measured six physicochemical parameters of the pond water (Secchi-disk transparency, pH, suspended solids, and concentrations of total nitrogen, total phosphorus, and chlorophyll *a*) once late in the same growing season when the macrophytes were surveyed. Water samples for chemical analysis were collected at a depth of 0.5 m at the approximate center of each pond, and field measurements were conducted at the same location (see Supplemental Material A for detailed methods). Unfortunately, water-quality data for 17 ponds were lost due to an accident, and thus water quality for each community type was summarized based on the remaining 352 ponds.

### Landscape and Local Variables

#### Selection of the Variables

We considered both natural environmental and anthropogenic factors in our analyses at the landscape and local scales. As natural environmental factors, we selected pond topographic configuration as a landscape-scale factor and pond surface area as a local-scale factor. The pond topographic configuration affects the macrophyte community composition (Kadono, 1998). Lentic water bodies that are low in the landscape tend to receive a smaller percentage of their incoming water from precipitation as compared to water bodies higher in the landscape (Kratz et al., 1997). Therefore, concentrations of dissolved organic carbon, vertical light penetration, pH, and conductivity base cations such as calcium and magnesium all may increase lower in the landscape (Kratz et al., 1997; Riera et al., 2000). Consequently, macrophytes that prefer nutrient-rich conditions, such as *Hydrilla verticillata* (L. f.) Royle and *Trapa japonica* Flerov, are found frequently in ponds located in flat lowlands, whereas those that prefer weakly acidic or oligotrophic conditions, such as *Brasenia schreberi* J. F. Gmel. and *Nymphaea tetragona* Georgi var. *tetragona*, are more likely to be observed in ponds located in hilly terrain. We also focused on surface area because it is often strongly associated with macrophyte community composition (e.g., Dodson et al., 2000; Jones et al., 2003; Akasaka and Takamura, 2012). As anthropogenic factors, we chose the proportions of urban area and paddy field (the prevailing agricultural type) surrounding the pond as landscape-scale factors and the proportion of natural shoreline covered by artificial embankment as a local-scale factor. These three anthropogenic factors have a strong impact on pond biodiversity (Akasaka et al., 2010; Kadoya et al., 2011).

#### Preparation of the Variables

We used 1:2500 orthorectified aerial photographs and 1:2500 digital geographic maps to determine the pond surface area, proportion of natural shoreline covered by artificial materials (mostly concrete; hereafter, “proportion of artificial embankment”), and geographic coordinates. Pond surface area and proportion of artificial embankment were ln-transformed before statistical analysis. For proportion of artificial embankment, a non-zero minimum value was added to all of the records before the transformation. To obtain data about degree of urbanization surrounding the ponds, we adopted a buffering approach (Pedersen et al., 2006) and used the proportion of urban terrain, including residential area and industrial area within 500 m of the pond’s edge, as a surrogate for urbanization. Likewise, proportion of paddy field within the same radius from the pond’s edge was used as a surrogate for agricultural land use. We chose this radius because previous findings indicated that the total species richness of macrophytes in a pond was best explained by the land-use proportion within 500 m (Akasaka et al., 2010). This choice was also supported by Cheruvelil and Soranno (2008), who reported that the floating and emergent macrophyte cover in a lake was best explained by road density within this radius. To determine the proportion of respective land-cover types around the pond, we used the most relevant vegetation and land-use map (scale 1:25000) from the Japan Integrated Biodiversity Information System^1^.

To quantify the topographic configuration of each pond, we adopted the TWI. Although TWI was originally proposed to represent the soil water content of a site (Moore et al., 1991), it can also be used to represent topographic configuration (or landscape position) (e.g., Svoray et al., 2004; Toyama and Akasaka, in press). TWI is calculated as:

$$\begin{array}{c} TWI\;=\;ln(A_{s}/tan\beta ), \end{array}$$

where *A*_s_ is the specific catchment area, defined from a digital elevation model, and β is the slope gradient. A low TWI value indicates that a pond is located in a hilly area, and a high TWI value indicates a relatively flat lowland area. To calculate TWI, we used a digital elevation map with 30-m resolution, which was interpolated from point elevation data with 50-m resolution provided by the Geospatial Information Authority of Japan. We used the minimum value of the TWI within a pond polygon as a representative value of the pond. All spatial data were analyzed using ArcGIS version 10.0 (ESRI, Redlands, CA, United States).

### Data Analyses

To evaluate the relative importance of landscape and local variables in determining the macrophyte community composition, we conducted a two-step analysis. Unlike a simple ordination analysis, this approach allows us to visualize a possible non-linear relationship. In addition, the results are easier to interpret for the non-specialist unfamiliar with interpreting biplot output, which is an important consideration when the goal is to support practical conservation planning.

In the first step, we clustered ponds according to their macrophyte community. To conduct the cluster analysis, we used the Jaccard distance measure and Ward’s linkage method. We identified the appropriate number of groups (we refer to each group as a “community type”) by examining the dendrogram while considering the need for parsimony and ease of interpretation. We tested for significant differences among the communities identified by the cluster analysis by using analysis of similarities (ANOSIM; Clarke, 1993), and used the Jaccard distance measure and 10,000 permutations in this analysis. Ponds with three or more macrophyte species (148 ponds) were subjected to the cluster analysis, whereas ponds with one or two macrophyte species (129) and those with no macrophytes (92) were treated as independent community types. These latter two community types were integrated with the community types identified by the cluster analysis in our subsequent analyses.

In the second step, we assessed the relationship between the occurrence of community types, including those identified in the cluster analysis and the two additional ones, and the five environmental factors. To do so, we modeled the occurrence of each community type using multinomial regression (Venables and Ripley, 2002). Landscape factors (TWI and proportions of urban area and paddy field) and local factors (pond surface area, proportion of artificial embankment) were used as the explanatory variables. We also added TWI^2^ as an explanatory variable, because preliminary analysis indicated that the relationship between the occurrence of community types and TWI can be unimodal. We constructed 64 (= 2^6^) candidate models that included all possible combinations of the explanatory variables. The model with the lowest value of Akaike’s information criterion (AIC; Burnham and Anderson, 2002) was selected as the best-fit model. We further ranked the relative contribution of each variable by calculating the corresponding Akaike parameter weight (Burnham and Anderson, 2002), which indicates the relative strength of the relationship between the variable and the response variable (i.e., community type) and represents the probability that the variable is included in the actual best model (Burnham and Anderson, 2002). Significance of each variable was also tested with a log-likelihood ratio test. We did not explicitly analyze the geographic location of each pond, because we found that Moran’s spatial autocorrelation coefficient (*I*) in the residuals on the probability of the best-fit model was low (*I* < 0.18) and most were non-significant (e.g., Van Langevelde and Wynhoff, 2009; Supplemental Material B). The magnitude of the correlations (|*r*|) between the explanatory variables was <0.54, except for the relationship between TWI and TWI^2^. Although we measured water-quality variables in 352 of the 369 ponds, these were not included as explanatory variables because of the well-reported effect of the five explanatory variables on the water-quality variables (e.g., Verhoeven et al., 2006); the inclusion of non-independent variables in a linear model as explanatory variables violates the fundamental assumption of dependence, which would not only disrupt parameter estimation but also complicate the interpretation of the responsible process and causal relationships.

We compared the six physicochemical parameters of pond water quality for each community type by using a general linear model. We set the link function of the general linear model as identity for pH and as log_10_ for the other parameters. For each parameter, we created models that included 52 possible combinations of the community types by merging the five community types identified by the cluster analysis (for details of the community types, see section “Classification of the Macrophyte Communities in Ponds”). The most plausible combination was determined by choosing the one with the lowest AIC (Burnham and Anderson, 2002).

All statistical analyses were conducted by using R version 3.4.2 (R Development Core Team, 2017). We used function *hclust* in package *stats* (R Core Team and Contributors Worldwide, 2017) for cluster analysis, *adonis* in package *vegan* (Oksanen et al., 2011) for ANOSIM, *multinom* in package *nnet* (Ripley, 2016) for multinomial regression, *dredge* in package *MuMIn* (Barton, 2018) to create and estimate all possible combinations of explanatory variables in the multinomial regression, *Anova* in package *car* (Fox and Weisberg, 2017) for likelihood ratio tests, and *lm* in package *stats* (R Core Team and Contributors Worldwide, 2017) for the general linear model.

## Results

### Classification of the Macrophyte Communities in Ponds

The 369 surveyed ponds captured a wide range of landscape and local features of ponds (**Table 1**). In total, we found 48 macrophyte species in the ponds, with the number per pond ranging from 0 to 14 (mean ± SD, 2.55 ± 2.67). However, 92 ponds (28%) had no macrophytes and 129 (40%) had only one or two species (**Table 2**). *Trapa japonica* was the most dominant macrophyte species in the surveyed ponds (observed in 174), followed by *Potamogeton octandrus* Poir. (58), *Nymphoides indica* (L.) Kuntze (58), and *Utricularia australis* R. Br. (55).

**Table 1:** Mean, standard deviation, and minimum and maximum values of the landscape and local variables.

Variables	Mean ± SD	Range
Landscape		
TWI	7.24 ± 1.15	5.12–11.32
Proportion of urban area	0.10 ± 0.11	0.00–0.73
Proportion of paddy field	0.44 ± 0.23	0.00–0.97
Local		
Pond surface area (m^2^)	8565 ± 8248	59–52,621
Proportion of artificial embankment	0.29 ± 0.30	0.00–1.00

*TWI, topographic wetness index.*

**Table 2:** Number of ponds, and mean ± standard deviation and range of number of species and of number of threatened species per pond in each community type discerned.

Community type	Number of ponds	Number of species		Number of threatened species		Frequently recorded species
		Mean ± SD	Range	Mean ± SD	Range	
Type I	45	5.67 ± 2.58	3–13	2.16 ± 1.52	0–7	*Nuphar saikokuensis* (0.67), *Brasenia schreberi* (0.67), *Utricularia australis* (0.64), *Nymphaea tetragona* var. *tetragona* (0.62), *Potamogeton octandrus* (0.44), *Trapa japonica* (0.42), *Potamogeton fryeri* (0.38)
Type II	32	4.12 ± 1.52	3–8	2.19 ± 1.45	1–7	*Nymphoides indica* (0.97), *Myriophyllum ussuriense* (0.56), *T. japonica* (0.50)
Type III	71	5.28 ± 2.39	3–14	1.52 ± 1.38	0–5	*T. japonica* (0.79), *Utricularia aurea* (0.41), *Hydrilla verticillata* (0.39), *P. octandrus* (0.38), *Najas oguraensis* (0.35)
Type IV	129	1.40 ± 0.49	1–2	0.33 ± 0.50	0–2	*T. japonica* (0.64)
Type V	92	0 ± 0	0	0 ± 0	0–0	
						
Total	369	2.55 ± 2.67	0–14	0.86 ± 1.28	0–7	Total 48 species, including 16 threatened species were observed

*The frequently recorded species observed in >35% of the ponds within a community type are shown. Numbers in parentheses indicate the proportion of ponds in the community type where the species occurred.*

**FIGURE 2:**
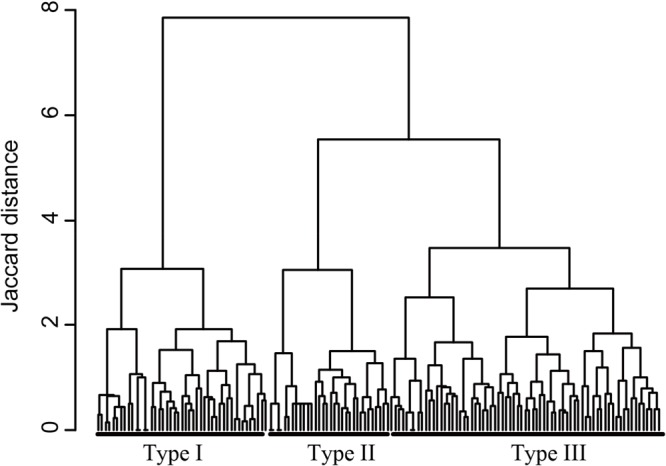
Cluster analysis divided the 148 ponds with at least three macrophyte species into community types based on the presence/absence data. **Table 2** provides statistical data for these community types, as well as those for Type IV (one or two macrophyte species) and Type V (no macrophyte species).

The cluster analysis divided the macrophyte communities with three or more species into three community types (**Figure 2**). Type I frequently harbored the species *Nuphar saikokuensis* Shiga et Kadono (observed in 67% of the Type I ponds), *B. schreberi* (67%), *U. australis* (64%), and *N. tetragona* (62%; **Table 2**). Type II most often included *N. indica* (97%), *Myriophyllum ussuriense* (Regel) Maxim. (56%), and *T. japonica* (50%). Type III most often contained *T. japonica* (79%), *Utricularia aurea* Lour. (41%), and *H. verticillata* (39%). These three community types differed significantly in their species compositions (pairwise ANOSIM, *P* < 0.001). We also defined Type IV (one or two species) and Type V (no macrophytes). Type IV frequently harbored *T. japonica* (64%; **Table 2**).

### Comparison of Water Quality Among the Community Types

The six physicochemical water parameters had a wide range of variation, suggesting that the surveyed ponds cover a broad portion of the eutrophication gradient. All the parameters showed significant differences among the five community types (**Table 3**). Overall, Type V without macrophytes occurred in more eutrophic waters, represented by low water transparency and high contents of total nitrogen, total phosphorus, chlorophyll *a*, and suspended solids, followed by Type IV, and then by the remaining three Types (I to III). Only Type V showed an alkaline pH, and Type I had the most acidic water (**Table 3**).

**Table 3:** Values of the six water-quality parameters for the five community types identified by cluster analysis.

Community	Total nitrogen (mg L^-1^)	Total phosphorus (mg L^-1^)	Transparency (m)	Chlorophyll *a* (g L^-1^)	Suspended solids (mg L^-1^)	pH
Type I	0.46 ± 0.19a	0.03 ± 0.02a	1.08 ± 0.38c	14.98 ± 17.68a	8.43 ± 8.00a	6.17 ± 0.52a
Type II	0.49 ± 0.17a	0.04 ± 0.03a	1.07 ± 0.54c	15.47 ± 16.90a	9.50 ± 7.18a	7.10 ± 0.61c
Type III	0.61 ± 0.50b	0.06 ± 0.08a	1.04 ± 0.65c	20.75 ± 33.24a	10.43 ± 10.44a	6.81 ± 0.72b
Type IV	0.72 ± 0.52b	0.09 ± 0.11b	0.76 ± 0.48b	32.29 ± 54.16b	13.03 ± 11.82b	6.95 ± 0.79b
Type V	1.04 ± 0.80c	0.14 ± 0.19c	0.53 ± 0.40a	62.73 ± 82.68c	20.72 ± 18.48c	7.91 ± 1.11d
						
Total	0.72 ± 0.57	0.08 ± 0.12	0.84 ± 0.53	32.95 ± 53.28	13.32 ± 13.16	7.08 ± 1.00

*Values are means ± standard deviation. In each column, values labeled with different letters differ significantly among the community types. Water-quality data for 6 ponds in Type V and 11 ponds in Type IV were not included for calculation.*

### Relationship Between Community Type and Landscape and Local Variables

Among the 64 candidate models that delineated the relationship between the community type in a pond and the six explanatory variables, the model that included all of the variables (including TWI^2^) was selected as the best-fit model, and all six variables were significant (**Table 4**). The ΔAIC of the second best-fit model (i.e., the difference in AIC from the that of the best-fit model), which employed all variables except proportion of artificial embankment, was 3.36, and those of the other 62 models were >7.7, indicating that all the variables that we selected play some role in determining the community type.

**Table 4:** Estimates of the best-fit multinomial regression model that describes the relationship between the probability of occurrence of a community type and the six landscape and local variables: TWI, TWI^2^, proportion of urban area around the pond, proportion of paddy field around the pond, pond surface area, and proportion of artificial embankment.

Community	Intercept	TWI	TWI^2^	Proportion of urban area	Proportion of paddy area	Surface area	Proportion of artificial embankment
Type I	–42.01	12.69	–0.88	–9.19	–4.28	–0.19	–0.08
Type II	–8.34	0.24	–0.02	–0.54	–0.01	0.70	–0.21
Type III	–8.33	0.97	–0.06	–3.02	–1.60	0.58	–0.22
Type IV	–15.98	4.76	–0.34	–1.36	0.18	0.01	0.01
						
Akaike parameter weight		0.99	0.99	0.98	1.00	1.00	0.84
*P*		0.000	0.000	0.034	0.001	0.000	0.022

*The statistical characteristics of the community types are summarized in **Table 1**. Akaike parameter weight was calculated to indicate the relative importance of the parameters in determining the community type that occurred in a pond. *P*-values were determined by likelihood ratio test.*

**FIGURE 3:**
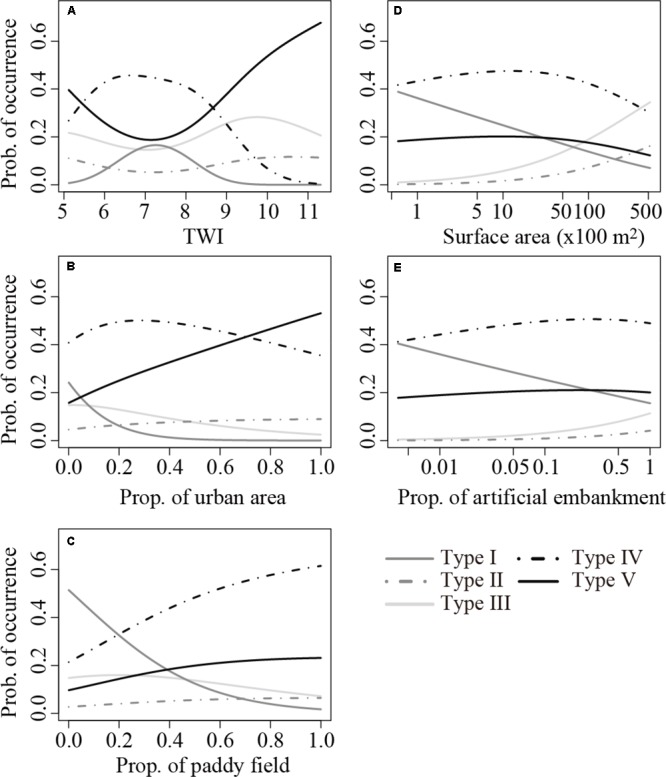
Relationships between the probability of occurrence of the five community types identified by the cluster analysis and **(A)** TWI, **(B)** proportion of urban area around the pond, **(C)** proportion of paddy field around the pond, **(D)** pond surface area, and **(E)** proportion of artificial embankment, predicted using the multinomial regression models shown in **Table 3**. Median values were assigned to the other dependent variables in the best model.

Transition among the three species-rich community types (Types I to III) was apparent among the two natural variables (i.e., TWI and surface area; **Figure 3**). The relationship between TWI and the probability of occurrence of Type I was unimodal, and the occurrence of Type I maximized at TWI = 7.3 (**Figure 3A**). After a slight initial decrease, an increase in TWI increased the probability of occurrence of Types II and III until peaking at TWI = 10.6 and 9.8, respectively, and both then gradually decreased. Increase in surface area decreased the probability of occurrence of Type I, whereas it increased the probabilities of occurrence of Types II and III (**Figure 3D**). In contrast, the three anthropogenic variables (i.e., proportions of urban area, paddy field, and artificial embankment) collectively decreased the probability of occurrence of Types I to III, except for slight increases in the occurrence of Types II and III with increasing proportion of artificial embankment (**Figures 3B,C,E**). The declining trend was sharpest for Type I for all three anthropogenic variables.

Overall, the probabilities of occurrence of Types IV and V (few or no macrophytes) increased with increases in the three anthropogenic variables (**Figures 3B,C,E**). In particular, a clear increase in the occurrence of Type V was observed as the proportion of urban area increased (**Figure 3B**), whereas that of Type IV was evident along with an increase in the proportion of paddy field (**Figure 3C**). Consequently, over 85% of the ponds were predicted to harbor either of these two community types when the proportion of urban area or paddy field equals 1. In contrast, the occurrence probability of these two types gently increased with increasing proportion of artificial embankment (**Figure 3D**).

## Discussion

The five landscape and local factors adopted in this study had a significant role in delineating the macrophyte community types in the 369 ponds (**Table 3**). Although these factors might represent only a subset of many potential determinants, our result nonetheless suggests that both natural environmental conditions and anthropogenic pressures influence the macrophyte community composition in the ponds and that the effects occur at both landscape and local scales. Therefore, in order to ensure a region’s pond biodiversity, our findings suggest the need for conservation at both spatial scales to maintain ponds with a variety of species compositions within a region, as noted in previous studies (e.g., Toivonen and Huttunen, 1995; Declerck et al., 2006; Rolon et al., 2012). Further, given that increases in the proportion of anthropogenic land use (urban area and paddy field) surrounding ponds had a greater impact on the occurrence of ponds with few or no macrophytes than an increase in the proportion of artificial embankment, our results call for greater emphasis on landscape-scale management actions (**Figure 3**), which need to be led by local and national agencies. This argument agrees with findings of Noble and Hassall (2015) who showed that ponds in urban landscapes have substantially lower plant biodiversity than would be expected at pristine sites. It contradicts studies on lakes that highlight the importance of local conditions in determining species composition (e.g., Capers et al., 2010; Alahuhta et al., 2013). This discordance can be attributed to a greater terrestrial-aquatic interchange of matter (Søndergaard et al., 2005), the greater proportion of land subjected to anthropogenic use around ponds (Søndergaard et al., 2005), and/or the exclusion from our model of water quality parameters, which is largely influenced by landscape-scale variables. As we have shown, understanding the relative importance of local and landscape-scale factors on the occurrence of community types in respective ponds provides valuable information for conservation as well as answering important ecological questions. We call for more studies focusing on ponds in different regions to evaluate the extent to which our findings are generalizable.

Our model showed clearly that high proportions of urban areas and paddy fields greatly increased the occurrence of Types IV and V (**Figure 3**), which were characterized by eutrophic water (**Table 4**). This result is supported by previous studies that showed negative impacts of both urbanization and extension of agricultural area on pond biodiversity (Declerck et al., 2006; Akasaka et al., 2010; Kadoya et al., 2011). However, these factors are predicted to lead to slightly different consequences: urbanization results in ponds with no macrophyte species, whereas the expansion of agricultural area allows a few macrophyte species to persist. This difference may be attributed to paddy fields reducing the concentration of nutrients such as nitrogen and phosphorus (Natuhara, 2013). Similarly, in Wisconsin, United States, the expansion of urban or agricultural areas around lakes led to differences in species composition (Mikulyuk et al., 2011). This suggests that the difference in consequences between urbanization and expansion of agricultural area that we detected in ponds might be common.

The two natural environmental factors drove the changes among communities of Types I to III, which had relatively high species richness (**Figure 3**). This suggests the importance of natural environmental conditions in determining the current macrophyte community composition, even in a region where the influence of anthropogenic pressures is dominant. Conservation and restoration targets for ponds in this region, therefore, should be set based on the detected relationship between the influential natural environmental conditions and the community type established.

When we differentiated the three community types with high species richness, TWI was an important determinant. Type I, which is predicted to emerge on hilly land, had the most acidic water (lowest pH; **Figure 3** and **Table 3**). The pH of water determines the form of the carbon source (i.e., CO_2_, ${\mathrm{HCO}}_{3}^{–}$, or ${\mathrm{CO}}_{3}^{2–}$) in the water, and the utilizable carbon source differs among macrophyte species (Maberly and Madsen, 1998). Thus, pH of the water is an important determinant of the emergence of macrophyte species in a community. Therefore, the transition of macrophyte communities along the TWI gradient might partly depend on pH changes related to the topographic configuration of the pond.

The pond surface area was negatively related to the probability of occurrence of Type I communities, but was positively related to the occurrence of Types II and III (**Figure 3B** and **Table 4**). This pattern indicates that Type I communities are less persistent in the face of pond enlargement than the other two macrophyte-rich communities. This might imply that small ponds in hilly areas should be enlarged carefully, if at all, to sustain the existing macrophyte community. Our data did not allow us to identify the factor(s) that clearly discriminate Type II from Type III, but water level fluctuation, maximum water depth, or both interactively could be responsible for the difference. *Nymphoides indica*, which requires exposed substrate for seedling establishment (Shibayama and Kadono, 2007), was observed in 97% of the Type II ponds, and 78% of this species’ occurrences within Types I to III ponds was concentrated in Type II. Therefore, further research on covariation among natural environmental factors, as well as social factors including the frequency of water use for irrigation and management intensity, which strongly influence the degree of water level fluctuation, will be necessary to clarify this issue.

### Implications for Conservation

To conserve regional pond biodiversity, previous studies noted that conservation actions must be taken not only on a focal pond but also in areas surrounding the pond (Kirchner et al., 2003; Williams et al., 2004; Declerck et al., 2006; Akasaka and Takamura, 2011, 2012). Although these studies indicated the need for conservation plans and actions focused on a larger spatial extent than an individual pond, they provided no basis to determine the actual spatial extent needed for conservation plans. In that sense, the transition of macrophyte community types along the gradient of topographic configuration that we detected provides a potential guideline, indicating that the spatial extent of a conservation plan should cover an area that includes the full gradient of topographic configurations (i.e., covering ponds from those in the upper hilly landscape to those in the lower flat landscape) in the region. In practice, we propose first zoning the area based on the topographic configuration (i.e., TWI value), and then selecting conservation priority ponds within each zone. Multiple ponds need to be conserved in each zone because β diversity of aquatic macrophytes (variation in species composition among ponds) is substantially larger than their α diversity (mean number of species in a pond) even if the topographic configuration is similar (Akasaka and Takamura, 2012). Based on our findings, when determining the zones, a TWI value around 7.3 is a plausible partitioning value to delineate a zone that targets macrophyte communities occurring in acidic, relatively oligotrophic water (i.e., Type I). Further research in different regions is required to determine widely applicable partitioning value(s).

Because urbanization and expansion of agriculture area strongly regulated the emergence of species-rich macrophyte communities, restriction of land development around the targeted ponds is a key issue for successful conservation of regional pond biodiversity. Such restrictions might be implemented by local and national governments, for example, by setting aside the areas for conservation or recommending eco-friendly rice farming while providing subsidies.

At a local scale, conservation action will be necessary to prevent further establishment of artificial embankments, which destroy macrophyte habitat (Nishihiro et al., 2006a). In some cases, however, to maintain the strength of a pond dike and to prevent disaster caused by collapse of the dike, artificial embankments are unavoidable. The use of the seed bank in the sediments (Nishihiro et al., 2006b) might mitigate the impacts caused by such construction and might restore the target macrophyte communities. Pond managers may want to integrate two or more small adjacent ponds to create a larger pond in order to increase the amount of water storage and reduce the labor cost for pond management (Takamura, 2012). To maintain existing macrophyte communities, however, the decision to enlarge a pond should be made carefully, especially when the pond is small and located in a less urbanized hilly area, because pond surface area significantly influences the macrophyte community. Although our results highlight the greater impact of anthropogenic land use as compared to that of artificial embankment, we believe that local action should continue to limit increases of artificial embankment because habitat provision is the basis of conservation as well as restoration. Joint efforts among entities that mainly work on different spatial scales (i.e., national and local governments, conservation groups, and pond managers) will be key to ensuring regional pond biodiversity.

## Author Contributions

MA conceived the ideas and designed methodology and writing of the manuscript. MA and SH collected and analyzed the data. All authors contributed critically to interpretation of the results and the drafts and gave final approval for publication.

## Conflict of Interest Statement

The authors declare that the research was conducted in the absence of any commercial or financial relationships that could be construed as a potential conflict of interest.

## Footnotes

1 http://www.biodic.go.jp/trialSystem/shpddl.html

## Abbreviations

TWI: topographic wetness index
