# Supplementary material for: Landscape- and Local-Scale Actions Are Essential to Conserve Regional Macrophyte Biodiversity
Source: Front Plant Sci. 2018 May 17;9:599. doi: 10.3389/fpls.2018.00599 (PMC5967199; doi:10.3389/fpls.2018.00599)
Supplement: Supplementary file 1 [file Data_Sheet_1.docx]

**Supplemental material A**

To describe the water quality for each macrophyte community type (described in subsection 3.1 in the Results section), we measured six physicochemical parameters of the pond water late in the same growing season when the macrophytes were surveyed. The Secchi-disc transparency and pH were measured near the center of the ponds; pH was determined with a glass electrode (HM-12P; DKK-TOA, Tokyo, Japan) at a depth of 0.5 m. Water samples for chemical analysis were collected at a depth of 0.5 m at the transparency and pH measuring point by filling a 2-L polypropylene bottle. After the sampling, the water bottles were kept cool and immediately transported to the laboratory for analysis. After persulfate digestion in disposable polycarbonate bottles held in an autoclave at 120 °C for 45 min, total nitrogen and total phosphorus concentrations were measured using the cadmium reduction method and the ascorbic acid method, respectively ([APHA, 1998](#_ENREF_4)). Suspended solids were collected on glass-fiber filters (Whatman GF/F; Whatman Incorporated, Clifton, NJ, USA) under a low vacuum and dried before weighing. Samples for determining the chlorophyll *a* concentration were collected onto GF/F filters, extracted into methanol (99% v/v), and measured spectrophotometrically ([Marker et al., 1980](#_ENREF_25)).

APHA (1998) Standard Methods for the Examination of Water and Wastewater. American Public Health Association, Washington, D.C.

Marker AFH, Nusch EA, Rai H, Riemann B (1980) The measurement of photosynthetic pigments in freshwaters and standardization of methods: conclusions and recommendations. Archiv Fur Hydrobiol 14:91-106

**Supplemental material B**


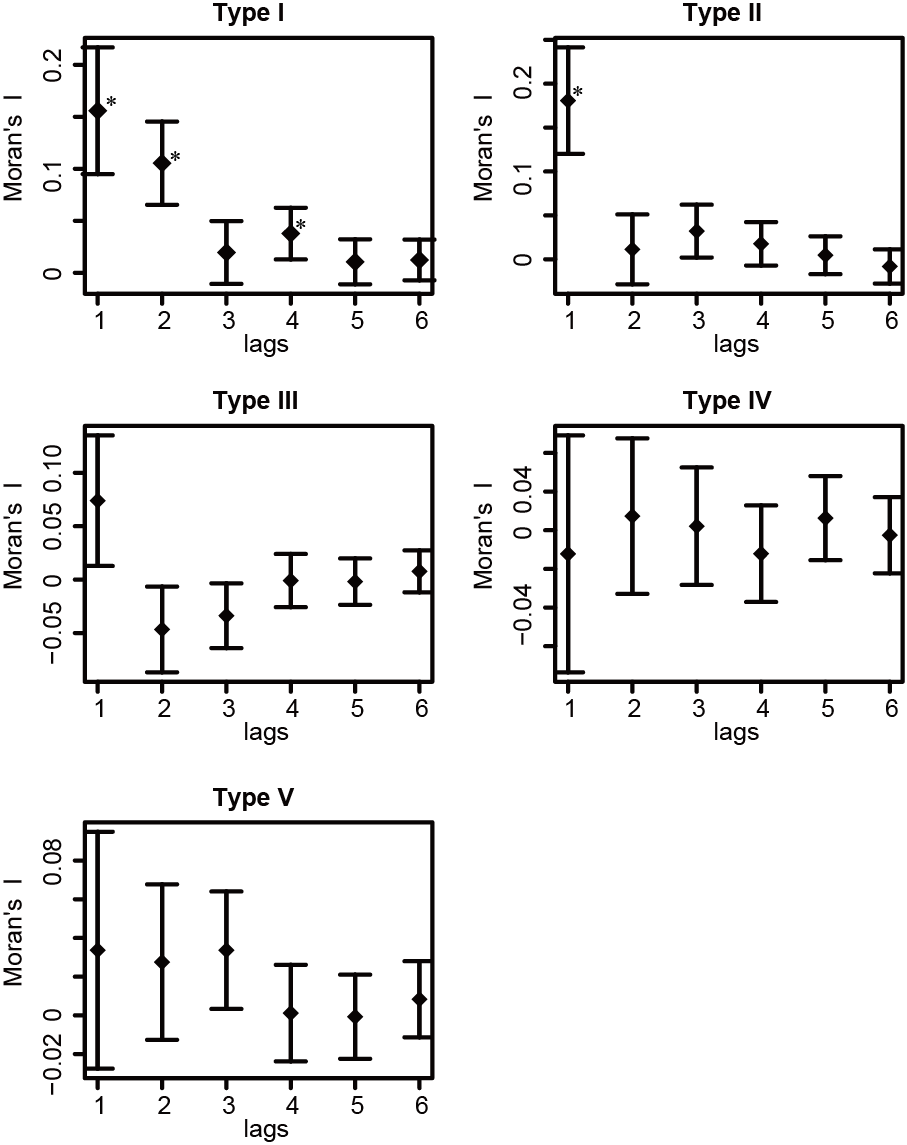


**Supplemental figure.** Moran’s *I* correlogram for residuals of the multinomial regression model. Asterisks indicate significance at *P* < 0.05 after Bonferroni correction.
